# Temporal discounting predicts procrastination in the real world

**DOI:** 10.1038/s41598-024-65110-4

**Published:** 2024-06-25

**Authors:** Pei Yuan Zhang, Wei Ji Ma

**Affiliations:** 1 Center for Neural Science, New York University, New York City, 10003 USA; 2 Department of Psychology, New York University, New York City, 10003 USA

**Keywords:** Temporal discounting, Procrastination, Real-world behavior, Motivation, Human behaviour

## Abstract

People procrastinate, but why? One long-standing hypothesis is that temporal discounting drives procrastination: in a task with a distant future reward, the discounted future reward fails to provide sufficient motivation to initiate work early. However, empirical evidence for this hypothesis has been lacking. Here, we used a long-term real-world task and a novel measure of procrastination to examine the association between temporal discounting and real-world procrastination. To measure procrastination, we critically measured the entire time course of the work progress instead of a single endpoint, such as task completion day. This approach allowed us to compute a fine-grained metric of procrastination. We found a positive correlation between individuals’ degree of future reward discounting and their level of procrastination, suggesting that temporal discounting is a cognitive mechanism underlying procrastination. We found no evidence of a correlation when we, instead, measured procrastination by task completion day or by survey. This association between temporal discounting and procrastination offers empirical support for targeted interventions that could mitigate procrastination, such as modifying incentive systems to reduce the delay to a reward and lowering discount rates.

## Introduction

In today’s world, achieving long-term goals, such as writing an article or developing complex software, demands sustained effort spanning days or months. These endeavors are crucial for both personal success and societal productivity, yet they often collide with the challenge of procrastination. Procrastination is prevalent; it chronically affects approximately 20% of the adult population^1^ and up to 70% of undergraduate students^2^. For instance, people delay filing their taxes until the last minute^3^. Researchers postpone until the last minute registering for academic conferences^4^ and submitting abstracts and papers^5^. College students commonly put off starting self-paced quizzes and find themselves rushing to complete them by the end of the semester^6–8^. The consequences of procrastination are profound, impacting individuals’ achievements and well-being. Procrastination results in lower salaries, shorter employment durations, a higher likelihood of unemployment^9^, and monetary loss^3^. Beyond these tangible effects, procrastinators frequently suffer from mental health challenges, including depression and anxiety, compounded by diminished motivation and low self-esteem^6,10,11^. Due to its high prevalence and high impact, procrastination is a problem of great societal importance.

The question arises: why do people procrastinate? Suppose you are a student who has to submit an assignment by a deadline. Initially, the utility of working on the assignment might be low because the deadline is far away, making work less appealing than alternative activities such as socializing. As a result, the student might delay working on the assignment until the utility of work exceeds the utility of socializing, which occurs as the deadline approaches. In line with this example, researchers in psychology and economics have, in different forms, hypothesized that temporal discounting is a mechanism underlying procrastination^12–16^. When faced with a task in its initial stages, where the eventual reward is distant, people temporarily discount the value of that future reward. As a consequence, the temporarily discounted future reward fails to provide sufficient motivation for people to start working until the deadline looms near.

This hypothesis predicts a positive correlation between the degree to which individuals discount future rewards and the extent of their procrastination. As far as we know, only three studies have attempted to test for this correlation^17–19^. Le Bouc and Pessiglione^17^ measured procrastination behavior in a survey completion task and found no evidence of a correlation. Sutcliffe et al.^18^ used a questionnaire to measure self-reported procrastination tendency and found no evidence of a correlation. Reuben et al.^19^ found a positive correlation in two real-world tasks that offered enhanced rewards as incentives for early completion. However, such incentives could be a confound because the actual correlation might be between temporal discounting and achievement motivation^20,21^. Indeed, the authors did not find a correlation when early completion incentives were removed in a third task. Two other studies^22,23^ appear to examine the relationship between temporal discounting and procrastination. They used a hyperbolic function to model the distribution of task completion time across individuals. The same function is commonly used to estimate temporal discount rates by modeling how future reward is discounted over time. However, these studies did not measure temporal discount rates, even though the same hyperbolic function was used.

In the present work, we used a novel measure of procrastination in a novel task to examine the association between temporal discounting and real-world procrastination behavior. It is common in the literature to use a single endpoint—task completion time—as a measure of procrastination^4,5,19,23–27^. However, individuals who complete a task at the same time can exhibit very different temporal patterns of work progress^28–30^. Some people maintain steady progress from beginning to end (steady working), whereas others make very little progress at the start and rush to complete their work on the very last day (rushing in the end). In order to better distinguish between such cases, we instead used a new metric to measure procrastination—Mean Unit Completion Day—that takes into account the entire time course of work progress.

We looked for a real-world task that satisfied three criteria. First, to rule out the potential confound in Reuben et al.^19^, no incentives should be given for early completion. Second, the task should measure the entire time course of work progress. This, in turn, requires that the task (a) has an unambiguous definition of a unit of work, (b) the completion time of each unit of work is measured, and (c) involves multiple units of work to establish a time course of work progress. Real-world tasks such as writing or taking an academic course often lack clearly defined units of work and are, therefore, not good candidates. Finally, an individual’s work progress in the task should not be affected by others.

A real-world task that satisfied these three criteria was the research participation requirement in the *Introduction to Psychology* course at New York University. To receive course credit, all enrolled students were required to participate in research studies for a total of 7 h before the end of the semester; the semester lasted a total of 109 days. This task was self-paced, granting students the autonomy to decide when to participate. All three criteria were met in this task. First, since course credit was independent of the time at which the research requirement was completed, no incentives were given for early completion. Second, a unit of work was clearly defined as 0.5 h because research participation opportunities involved a time commitment of 0.5, 1, 1.5, or 2 h. The vast majority (91.2%) of participation opportunities took 0.5 h or 1 h. The date of each research participation was documented in the New York University Sona System and was accessible to the system administrator. Students needed to participate multiple times to fulfill the 7-h requirement. All students participated at least six times, with a median of 10 times. Last, research participation opportunities were plentiful: an average of 15 h per student. Thus, there was no need for students to compete for these opportunities, and each student’s work progress could reasonably be assumed to be independent of that of others. In contrast, if research participation opportunities are limited, whether a student can participate in a study on a certain day depends on whether other students have already taken that opportunity. In this case, a student’s work progress will be dependent on others due to the need to compete for scarce resources.

To estimate the degree of reward discounting, two weeks after the semester ended, we invited all students who had been enrolled in the course to participate in our online study that included a delay discounting task. Participants were asked to indicate their monetary preferences between smaller but sooner rewards and larger but delayed rewards (Fig. 1A). We used a widely adopted choice set designed to capture a broad range of discount rates^31–37^. The delays in the choice set ranged from 1 to 180 days, comparable to the 109-day research participation task. Moreover, this task was designed to be incentive-compatible, in contrast to the hypothetical nature of rewards in the previous studies^17,19^.

The secondary objective of our study was to examine the relationship between risk attitude and behavioral procrastination. By postponing the research participation until the end of the semester, students face an increased risk of not being able to complete the research participation requirement, particularly when considering other competing obligations near the end of the semester, such as final exams. Consequently, procrastination in the research participation task can be viewed as a risk-seeking behavior. A prior study^38^ found no evidence of a correlation between the risk attitude measured by the Domain-Specific Risk-Taking (DOSPERT) scale^39^ and self-reported procrastination tendency measured by the Lay Procrastination Scale^40^. In this real-world task, we examined the relationship between people’s risk attitude and procrastination behavior. To estimate participants’risk attitude, we included three measures in our online study: the incentive-compatible risky-choice task (Fig. 1B), which assessed risk attitude primarily within the financial domain^41,42^; the DOSPERT scale, which assessed risk attitude across five domains (ethical, health/safety, recreational, financial, and social), and a set of custom-designed questions that assessed risk attitudes specifically toward postponing research participation in our real-world task (see “Methods”).

## Results

**Figure 1:**
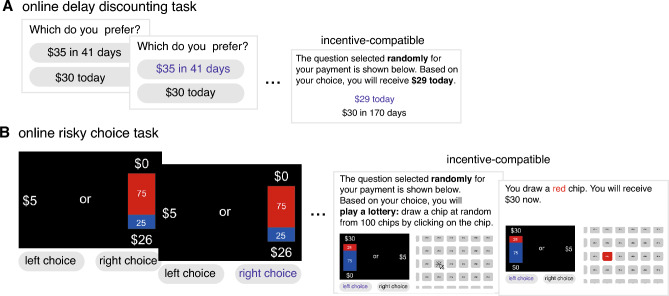
Tasks. (**A**) Online delay discounting task. In each trial, participants were first presented with two options: a smaller immediate reward today and a larger reward with a delay of several days, and then they indicated their preference by choosing one. At the end of the task, their choice of one randomly selected trial will determine the payment amount and the day of delivery. (**B**) Online risky choice task. In each trial, participants were first presented with two options: receiving $5 for sure and participating in a lottery where they had a chance to win a larger amount with a certain probability, otherwise receiving $0, and then they indicated their preference by choosing one. At the end of the task, the choice of one randomly selected trial will determine the payment. If participants chose the sure bet, they would receive $5. However, if they chose the lottery, they would play it by randomly drawing a chip from 100 chips. As the task was conducted online, we gave participants a visual aid of the chip-drawing process by displaying 100 chips and instructing them to click on a chip to simulate the random draw. After clicking, the color of the chip will be revealed, and the payment will be based on the result of the lottery.

### Participants

Participant inclusion was determined as follows. First, to ensure that our measures of procrastination would not be confounded by the total number of work units completed, we selected the participants who met their 7-h requirement and did not continue to do more research sessions beyond the requirement. This resulted in a total of 93 participants. Second, we applied pre-registered exclusion criteria to the delay discounting task. We excluded 9 participants who either failed two or more of the five attention check questions or consistently chose one option. Finally, we conducted a quality control procedure to ensure that participants were not responding randomly (see “Methods”). No additional participants were excluded based on this procedure. This left us with a final sample of 84 participants to test the relationship between temporal discounting and procrastination and the convergent validity of our measurement of procrastination (53 female, 28 male, two non-binary, one unknown; $19.4 \pm 1.4$ years old). To test the relationship between risk attitude and procrastination, we applied similar exclusion criteria and quality control to the risky-choice task (see “Methods”), leaving us with a sample of 91 participants (56 female, 31 male, three non-binary, one unknown; $19.3 \pm 1.8$ years old).

### Characterizing individual variability in procrastination

**Figure 2:**
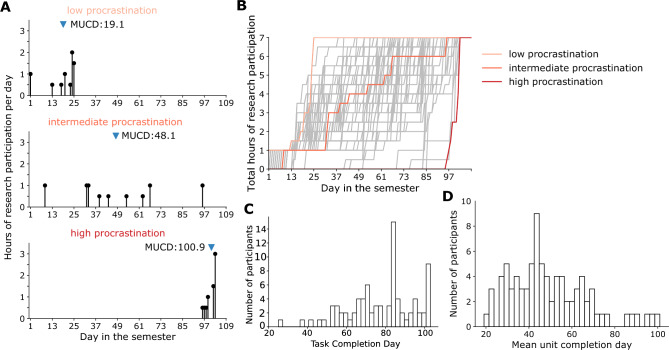
Procrastination in the real world. (**A**) Examples of time courses of work progress, with blue triangles marking the Mean Unit Completion Day (MUCD). Top: a low procrastinator who started on the first day and finished early. Middle: an intermediate procrastinator who worked steadily throughout the semester. Bottom: a high procrastinator who rushed to complete the task in the last two weeks of the semester. (**B**) Time courses of cumulative work progress for all the participants, with the three examples from (**A**) highlighted. (**C**) Histogram of task completion day. (**D**) Histogram of MUCD.

In the research participation task, we found that the time course of work progress differed greatly between individuals, ranging from participants who started and finished early to those who waited until the last two weeks of the 109-day period (Fig. 2A). The cumulative progress curves across all the participants clearly show this high individual variability (Fig. 2B).

There are many ways of summarizing a time course of work progress, some of which have been used in previous papers. Perhaps the most obvious summary statistic is task completion time^17,19,24–27^. In our task, the distribution of task completion day is wide, ranging from 25 to 103 days ($M=77.5$, $SD=17.2$) (Fig. 2C). Another metric is the amount of work (in our task, the number of hours of research participation) completed in the last third of the semester^6^ ($M=1.7$, $SD=2.0$) (Supplementary Fig. S1A). Furthermore, one could use task starting day^25,27,43,44^. In our task, however, students were asked by the instructor to complete the first research participation in the first two weeks. This separate deadline makes the task starting day somewhat contaminated as a measure of procrastination in the overall research participation task. Nevertheless, we show the distribution of task starting day in Supplementary Fig. S1B.

The above metrics take into account only a single point or partial segment of the time course of work progress. Next, we turn to metrics that consider the entire time course of work progress. We introduce a novel metric, Mean Unit Completion Day (MUCD), as the average completion day of all work units, with each work unit defined in this task as 0.5 h of research participation (see the formula in the Supplement). MUCD had a wide distribution, ranging from 19.1 to 100.9 ($M = 49.6$, $SD= 18.2$), further demonstrating the high level of individual variability in procrastination (Fig. 2D).

We assessed the convergent validity of MUCD by testing whether MUCD in the research participation task is associated with self-reported procrastination in general academic situations. We measured participants’general academic procrastination tendencies with the widely used Procrastination Assessment Scale for Students (PASS)^6^. Participants were asked to report the frequency with which they procrastinated on tasks such as writing term papers, studying for exams, and four other academic scenarios. Our findings revealed a moderate positive correlation between MUCD and PASS score (Pearson $r=0.42$, $p<0.001$), which provides support for the convergent validity of our measure.

Two other metrics are closely related to MUCD. The first is the day of the halfway point of the work^7^, which is the median of the time course of work progress ($M = 50.5$, $SD= 22.4$) (Supplementary Fig. S1C). The second is the area under the cumulative progress curve^30^; however, we prove here that this metric is mathematically equivalent to MUCD (see proof in the Supplement).

Besides MUCD, the other metrics were also correlated with the PASS score, suggesting the convergent validity of these measures (task completion day: $r=0.31$, $p=0.005$; hours in the last third of the semester: $r=0.41$, $p<0.001$; task starting day: $r=0.36$, $p<0.001$; day of the halfway point: $r=0.42$, $p<0.001$;). All metrics considered were correlated with each other (see Supplementary Table S1). All metrics were preregistered, except for task starting day (because of the potential confound of a different deadline) and area under the cumulative progress curve (because of the mathematical equivalence).

### Discount rate correlates with behavioral procrastination quantified by MUCD but not task completion day or survey-based measure

**Figure 3:**
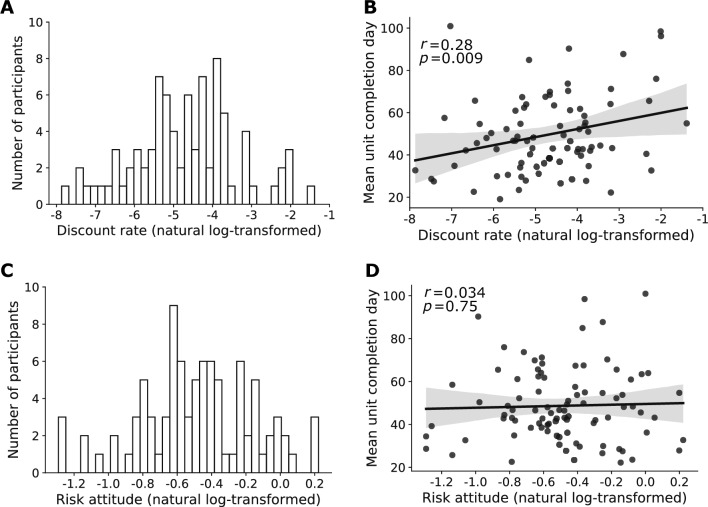
Procrastination correlates with discount rate but not risk attitude. (**A**) Histogram of the natural log-transformed discount rate estimated from the delay discounting task. (**B**) Correlation between MUCD and the natural log-transformed discount rate. (**C**) Histogram of the natural log-transformed risk attitude parameter estimated from the risky-choice task by fitting a power utility model. (**D**) Correlation between MUCD and the natural log-transformed risk attitude estimated from risky-choice task.

Turning to our main question, we examined the correlation between temporal discounting and procrastination. We estimated individual temporal discount rates through the incentive-compatible delay discounting task. We fit a hyperbolic choice model to the choice data of each participant. The discount curves were well characterized by hyperbolic functions (goodness of fit: $M=0.73$, $SD=0.14$). We found high variability (Fig. 3A): the natural log-transformed discount rate ranged from $-7.87$ (equivalent to a 1.14% discount of reward value after 30 days) to $-1.39$ (an 88.2% discount of reward value after 30 days). We found a positive correlation between the discount rate and MUCD ($r=0.28$, $p=0.009$) (Fig. 3B). In addition, after controlling for age and gender, the discount rate was still positively associated with MUCD ($\beta =3.6$, $SE=1.4$, $t(78)=2.53$, $p=0.013$).

We found that (a) day of the halfway point and (b) hours in the last third semester both correlated with the discount rate ($r=0.28$, $p=0.009$; $r=0.24$, $p=0.030$, respectively), but metric (c) task completion day or (d) task starting day did not ($r=0.21$, $p=0.061$; $r=0.18$, $p=0.098$, respectively). These results held true after we controlled for age and gender (day of the halfway point: $\beta =4.3$, $SE=1.7$, $t(78)=2.52$, $p=0.014$; hours in the last third semester: $\beta =0.33$, $SE=0.16$, $t(78)=2.04$, $p=0.044$; task completion day: $\beta =2.4$, $SE=1.3$, $t(78)=1.80$, $p=0.077$; task starting day: $\beta =2.8$, $SE=1.8$, $t(78)=1.57$, $p=0.12$). One interpretation of these findings is that measures based on the time course of work progress have greater statistical power than measures based on an endpoint.

We found no correlation between the discount rate and the PASS score ($r=0.21$, $p=0.056$; after we controlled for age and gender: $\beta =0.088$, $SE=0.053$, $t(78)=1.65$, $p=0.10$), highlighting the advantage of behavioral measures of procrastination over survey-based measures.

As an exploratory analysis, we tested if impulsivity, self-control, or perfectionism mediate the correlation between temporal discounting and procrastination. Details are provided in the Supplement.

### No evidence of a correlation between risk attitude and behavioral procrastination

To estimate participants’risk attitude, we employed three approaches: the incentive-compatible risky-choice task (Fig. 1B), the Domain-Specific risk-taking (DOSPERT) scale^39^, and a set of custom-designed questions that assessed risk attitudes specifically toward postponing research participation in this research participation task: The first question measured participants’perception of the risk associated with not being able to fulfill the research participation requirement by postponing it until the end of the semester, while the last two measured the level of aversion to that risk (see “Methods”).

We fitted a power utility model to the individual choice data from the risky-choice task. We found high variability in the risk attitude parameter (Fig. 3C): the natural log-transformed risk attitude ranged from −1.29 to 0.22. We found no evidence of a correlation between risk attitude and behavioral procrastination in this research participation task characterized by MUCD (Fig. 3D), day of the halfway point, hours in the last third semester and task completion day ($r=0.034$, $p=0.75$; $r=0.10$, $p=0.35$; $r=0.068$, $p=0.52$; $r=-0.064$, $p=0.55$, respectively).

Similarly, we did not find a significant correlation between procrastination and risk attitude measured by the DOSPERT scale across five domains (correlation between MUCD and the mean DOSPERT score: $r=-0.12$, $p=0.25$). Specifically, we did not find a correlation between MUCD and risk-taking in the ethical domain ($r=-0.081$, $p=1.0$), in the financial domain ($r=0.13$, $p=0.83$), in the health/safety domain ($r=0.012$ $p=1.0$), in the recreational domain ($r=-0.16$, $p=0.60$), or in the social domain ($r=-0.022$, $p=1.0$) (corrected using the Holm-Bonferroni method). Additionally, we did not find a correlation between procrastination and risk perception across five domains measured by the DOSPERT-Risk Perception subscale (correlation between MUCD and risk perception in the ethical domain: $r=0.011$, $p=1.0$, in the financial domain: $r=-0.24$, $p=0.11$, in the health/safety domain: $r=-0.065$, $p=1.0$, in the recreational domain: $r=-0.035$, $p=1.0$, or in the social domain: $r=0.025$, $p=1.0$. (corrected using the Holm-Bonferroni method))

Next, we analyzed the questions custom-designed to measure risk attitudes specifically toward postponing research participation. In terms of risk perception (the first question), participants strongly agreed that postponing research participation until the end of the semester increased the risk of not being able to fulfill the requirement (ratings ranging from strongly disagree (1) to strongly agree (7); $Median = 7$; $Mean = 6.2$; $SD = 1.2$). However, we did not find evidence of a correlation between risk perception and procrastination characterized by MUCD, day of the halfway point, hours in the last third semester, or task completion day ($r=-0.16$, $p=0.14$; $r=-0.14$, $p=0.18$; $r=-0.19$, $p=0.07$; $r=-0.12$, $p=0.27$, respectively). The results were qualitatively the same for risk attitude (average score across the second and third questions): Participants reported a high level of aversion to the risk of not fulfilling the requirement due to postponing the research participation ($Median = 5$; $Mean = 4.8$; $SD = 1.3$). However, we did not find evidence of a correlation between risk attitude and procrastination characterized by MUCD, day of the halfway point, hours in the last third semester, or task completion day ($r=-0.094$, $p=0.37$; $r=-0.13$, $p=0.21$; $r=-0.015$, $p=0.89$; $r=-0.090$, $p=0.40$, respectively).

### Self-reports of procrastination behavior

At the end of our online study, participants answered custom-designed questions about their views on procrastination in the research participation task. For example, they were asked how satisfied they were with how they allocated their time over the semester to fulfill the requirement, their attribution of procrastination, and their top-rated reasons for procrastination (see the Supplement for results). Here, we highlight one result: participants were aware of their own level of procrastination in research participation. Participants were asked to rate their procrastination level from not at all (1) to an extreme extent (5) in fulfilling the research participation requirement. We found that the rating of their own procrastination level in research participation positively correlates with their behavioral level of procrastination characterized by MUCD ($r=0.68$, $p<0.001$). This suggests that participants were aware of their own level of procrastination in the task.

## Discussion

We have presented evidence for an association between reward discounting and procrastination behavior in a long-term real-world task. This suggests that temporal discounting is a potential cognitive mechanism underlying procrastination.

Why did prior studies^17–19^ fail to find a correlation between temporal discounting and procrastination? One reason might be that the choice sets they used might not have allowed for estimating the discount rate with the same precision as ours. Another reason might be that their delay discounting task was not incentive-compatible. Finally, their measurement of procrastination might not be as precise as ours. Sutcliffe et al.^18^ did not employ a behavioral measure of procrastination; instead, they used a questionnaire. When we applied a similar questionnaire method, no evidence of a correlation was found. The other two studies^17,19^ measured behavioral procrastination but limited their metrics to the task completion day, as they did not measure the entire time course of work progress. By contrast, we measured the entire time course of work progress and computed fine-grained metrics of procrastination, such as MUCD. This approach might provide greater statistical power than simply using the task completion day as a metric, which, when we applied it, also resulted in no evidence of a correlation. Alternatively, it is possible that stronger and weaker discounters truly do not differ in task completion day but only in how they allocate their time before completion. Future work will need to distinguish these two possibilities.

The observed association between temporal discounting and procrastination suggests two types of interventions to reduce procrastination: one is changing the incentive system, and another is reducing procrastination via lowering discount rates. First, regarding the incentive system, one might reduce procrastination by decreasing the delay in receiving a reward. While previous work has shown that adding immediate rewards to the original incentive environment enhances persistence^45,46^ and reduces procrastination^47^, it remains unclear whether these effects are due to the increased reward magnitude or to a change in reward timing. Future research should disentangle these two factors and test the effect of decreasing the delay to a reward.

Second, procrastination can be reduced by lowering discount rates. The most promising ways to lower discount rates are episodic future thinking and mindfulness-based training/acceptance-based training^48,49^. Mindfulness-based training has been shown to be effective in reducing procrastination^50–53^, but no studies have tested the effect of episodic future thinking on procrastination. One study showed a negative association between episodic future thinking and procrastination^54^. However, the effectiveness of episodic future thinking as an intervention remains to be studied. Future studies should test this intervention using a randomized control trial. Furthermore, future studies could test whether a reduced discount rate mediates the effectiveness of reducing procrastination through episodic future thinking or mindfulness-based training. In addition, the effects of these interventions could vary among individuals with different discount rates (e.g., healthy controls versus clinical populations^55^). For example, people with ADHD might be more sensitive to interventions that reduce procrastination by lowering discount rates^56^.

Limitations of our work include the use of a WEIRD^57^ sample of NYU undergraduates and the use of a non-academic task. Future work should generalize to more diverse global samples and non-academic tasks. Moreover, it is possible that students frame the outcome of the research participation task as avoiding losses (“if I don’t fulfill the requirement, I might lose the credit for the course”) instead of as pursuing gains (“if I fulfill the requirement, I will get the credit for the course”)^58^. Future research could test if the discounted value of a future loss is also associated with procrastination.

More work is needed to understand the mechanisms underlying the observed association between temporal discounting and procrastination. First, it is possible that the association is due to a common cause. One candidate common cause is time perception^59,60^. The intuition is that a person who perceives a short period as longer tends to procrastinate because they think they have more time. The same person could be more likely to choose an immediate reward over a delayed reward because they perceive the delay to be longer.

Second, previous authors have distinguished between two forms of delay associated with procrastination: a delay in making a decision and a delay in implementing an action^61,62^. In our case, these would translate to choosing which research study to participate in and actually participating in it, respectively. Our empirical measure of procrastination does not distinguish between these two forms of delay. It would be interesting to test which form of delay is mainly responsible for the observed association between temporal discounting and procrastination.

In summary, we provided the first empirical evidence of an association between temporal discounting and procrastination in the real world. This finding not only suggested a potential cognitive mechanism underlying procrastination but also suggested a new approach to characterizing procrastination behavior and new interventions.

## Methods

### Procedure

We sent email invitations with a link to our online study to all the students enrolled in the 2021 *Introduction to Psychology* course two weeks after the semester ended. In the email, we provided a broad description of the study’s aim, investigating the factors influencing student research participation. We did not disclose the specific focus of the study on procrastination.

Our online study included a delay discounting task to estimate the discount rate, a risky choice task, the Domain-Specific risk-taking (DOSPERT) scale^39^, and a set of custom-designed questions to estimate risk attitude jointly. It also included the Procrastination Assessment Scale for Students (PASS)^6^ to test convergent validity. For exploratory analysis (details in the Supplement), we included surveys and custom-designed questions to address several aspects of procrastination in the research participation task. We included the Barratt Impulsivity Scale^63^, the Brief Self-Control Scale^64^, and perfectionism scales^65,66^ to test their association with behavioral procrastination and whether they mediate the correlation between temporal discounting and procrastination. We also included custom-designed questions aimed at assessing participants’awareness of their procrastination levels in the task and their satisfaction with the way they allocated their time over the semester to fulfill the research participation requirement. Additionally, we included the Regret Elements Scale^67^ to test whether high procrastinators regret the way they allocated their time to fulfill the requirement, the Causal Dimension Scale^68^ to test attribution of procrastination and success in fulfilling the requirement, and the Reasons for Procrastination Scale^6^ to identify the top-rated reasons for procrastination. All the tasks and surveys were counterbalanced in order, and tasks were presented before the surveys.

All participants gave informed consent prior to participating. Participants were compensated with $5 for their participation and had the opportunity to earn a bonus of up to $66 based on their choices during the tasks. This study was approved by New York University’s Institutional Review Board (IRB-FY2020-4262), and all experiments were performed in accordance with relevant guidelines and regulations. This study was pre-registered on Open Science Framework (https://osf.io/4sxrw).

### Participant inclusion

The sample size of the online study was 194, which was 25.9% of the students who had been enrolled in the *Introduction to Psychology* course. To ensure that our measures of procrastination would not be confounded by the total number of work units completed, we only included the subset of participants who did not continue to do research sessions after they had met their 7-h requirement. For example, we would include a participant who, after completing 6.5 h, did a final research session to meet the requirement. However, we would exclude one who, after completing 7 h, did an additional session that was not required. This resulted in a total of 93 participants. Of the remaining 101 participants, 80 continued to do research sessions beyond the 7-h requirement, potentially to earn extra credit. The remaining 21 completed fewer than 7 h; in some cases, this was because they completed an alternative assignment (i.e., writing critique papers).

To test the hypothesis of correlation between temporal discounting and procrastination, out of 93 participants, we excluded 9 who either failed two or more of the five attention check questions or consistently chose one option in the delay discounting task, as that would make it impossible to determine their discount rate. To ensure that participants were not responding randomly, we conducted a quality control procedure^69^. We verified that participants’responses were influenced by task-relevant variables. This involved fitting a logistic regression model that included as predictors the immediate amount, the delayed amount, the delay, and the squares of these variables to each participant’s responses. The goodness of fit of the model was assessed using the coefficient of discrimination, and any participant with a value below 0.2 was considered a random respondent. No participants were excluded as random respondents. This left us with a final sample of 84 participants.

To test the hypothesis of correlation between risk attitude and procrastination, out of 93 participants, we excluded two subjects who either chose the objectively worse option in two or more of seven attention check trials or who consistently chose one option in the risky choice task, as that would be impossible to determine their risk attitude. Similarly to the delay discounting task, we conducted a quality control procedure to ensure that participants were not responding randomly. We verified that participants’ responses were influenced by task-relevant variables. This involved fitting a logistic regression model that included as predictors the winning amount of the lottery, the probability of winning the lottery, and the squares of these variables to each participant’s responses. The goodness of fit of the model was assessed using the coefficient of discrimination, and any participant with a value below 0.2 was considered a random respondent. No participants were excluded as random respondents. This left us with a final sample of 91 participants.

### Delay discounting task

The delay discounting task consisted of 51 self-paced trials in which participants chose between receiving a smaller amount of money immediately or a larger amount after a specific number of days. The immediate reward ranged from $10 to $34, while the delayed reward was fixed at $25, $30, or $35, with delays ranging from 1 to 180 days. This choice set was designed to capture a broad range of discount rates evenly distributed in log space within the range of $$[-1.6, -8.4]$$. It was adapted from Kirby’s choice set^31^ and has been widely used in the temporal discounting literature^31–37^. To minimize any potential biases, we counterbalanced the position of the immediate reward on the screen (up or down). Additionally, we included five attention check trials in which participants were asked to choose between a larger immediate amount of money and a smaller amount with a delay.

We estimated temporal discount rates by fitting a hyperbolic choice model to the choice data of each participant. The utility of each option (immediate or delayed) is given by: $U=\frac{v}{1+kD}$, where *U* is the subjective discounted value, *v* is the monetary reward, *D* is a delay in days, and *k* is the individual discount rate. We used the softmax function to generate choice probabilities from option values.

$$\begin{aligned} \text {Pr}_{\text {delayed}} = \frac{1}{1 + e^{-\beta (U_{\text {delayed}} - U_{\text {immediate}})}} \end{aligned}$$

where $\text {Pr}_{\text {delayed}}$ is the probability that the participant chose the delayed option on a given trial, and $\beta$ is the inverse temperature, which captures the stochasticity of the choice data. We used maximum-likelihood estimation to estimate the model parameters. We calculated the average goodness of fit as one minus the ratio between the log-likelihood of the model and that of a random-response model.

### Risky-choice task

The risky-choice task consisted of 57 trials, each involving a choice between receiving $5 for sure and participating in a lottery where participants had a chance to win a larger amount with a certain probability, otherwise receiving $0. For example, one trial presented participants with a choice between $5 for sure and a 25% chance of winning $16 or a 75% chance of receiving $0. The larger amounts ranged from $6 to $66, and we used three different winning probabilities: 25%, 50%, and 75%. This choice set was adapted from a previous study^42^. To minimize any potential biases, we counterbalanced the position of the sure-bet option on the screen (left or right) and the associated color of the larger amount (blue or red). Additionally, we included seven attention check trials that presented participants with a choice between $5 for sure and a certain chance of receiving $4 or $5.

To help participants better understand the probabilities involved, the instructions included a visual representation of the choices. Each lottery image depicted a physical bag containing 100 poker chips, including red and blue chips. The size of the colored area and the number written inside indicated the number of chips of each color in the bag. The process of randomly drawing a chip was referred to as “playing the lottery.”

We estimated individual risk attitudes by fitting a power utility model to the trial-by-trial choice data. In this model, the utility of each option (safe or lottery) is given by $U=pv^\alpha$, where *v* is the dollar amount, *p* is the probability of winning, and $\alpha$ is the individual’s risk attitude. A participant with $\alpha >1$ is considered risk-seeking, $\alpha =1$ is considered risk-neutral, or $\alpha <1$ is considered risk-averse. Like in the delay discounting task, we used the softmax function to generate choice probabilities from option values.

$$\begin{aligned} \text {Pr}_{\text {lottery}} = \frac{1}{1 + e^{-\gamma (U_{\text {lottery}} - U_{\text {safe}})}} \end{aligned}$$

where $\text {Pr}_{\text {lottery}}$ is the probability that the subject chose the lottery on a given trial, and $\gamma$ is the inverse temperature which captures the stochasticity of the choice data. We used maximum-likelihood estimation to estimate the model parameters.

### Incentive compatibility

Both the delay discounting task and the risky choice task were incentive-compatible. Participants were offered a bonus: at the end of the study, their choice from a randomly selected trial in either the delay discounting task or the risky choice task determined the amount of this bonus. The bonus was provided as an electronic Amazon Gift Card. If the one randomly selected trial is from the delay discounting task, the timing of receiving the bonus depends on the chosen option. Specifically, for payment today, participants received the gift card on the same day. For delayed payments, participants received the gift card at a time corresponding to the delay associated with their chosen option. If the one randomly selected trial is from the risky choice task, if participants chose the sure bet in the selected trial, they would receive $5. However, if they chose the lottery, they would engage in the process of drawing a chip at random from a set of 100 chips. As the task was conducted online, we provided participants with a visual aid of the chip-drawing process. We displayed 100 chips and instructed participants to click on a chip to simulate the random draw. After clicking, the color of the chip would be revealed to indicate the result of the lottery.

### Custom-designed questions to measure risk attitude toward postponing research participation

We designed three questions to measure the participants’risk perception and risk attitude regarding delaying their research participation until the end of the semester. The first question measured their perception of the risk associated with not being able to fulfill the research participation requirement by postponing it until the end of the semester. Participants were asked to rate their agreement with the statement from strongly disagree (1) to strongly agree (7): “I believe that postponing one’s research participation until the end of the semester increases the risk of not being able to fulfill the research participation requirement.”

The second and third questions aimed to measure the extent of aversion to the risk of not fulfilling the requirement due to delaying research participation until the end of the semester. Participants were asked to rate their agreement with the two statements from strongly disagree (1) to strongly agree (7): “The increased risk of not being able to fulfill the research participation requirement due to postponing the research participation was motivating and exciting for me” (with reversed key) and “The increased risk of not being able to fulfill the research participation requirement due to postponing the research participation was stressful or anxiety-inducing for me.”

### Supplementary Information

Supplementary Information.

## Data Availability

Experimental stimuli, anonymized data, and scripts for analysis are available through the Open Science Framework (https://osf.io/z548y/).
