# Supplementary material for: Temporal discounting predicts procrastination in the real world
Source: Sci Rep. 2024 Jun 25;14:14642. doi: 10.1038/s41598-024-65110-4 (PMC11199680; doi:10.1038/s41598-024-65110-4)
Supplement: Supplementary file 1 — Supplementary Information. [file 41598_2024_65110_MOESM1_ESM.docx]

**Supplementary Information for**

**Temporal discounting predicts procrastination in the real world**

Pei Yuan Zhang, Wei Ji Ma

Correspondence to Pei Yuan Zhang

Email: pz580@nyu.edu

**Metrics of procrastination**

Steel et al. (2018)[^1^](https://paperpile.com/c/AeZq7C/KA9f) proposed the area under the cumulative progress course curve (AUC) as a measure of procrastination. We demonstrate here that AUC is a linear transformation of MUCD. We consider a general situation in which a person completes *N* units of work over *T* days. We denote the number of units completed per day by $x_{1}, x_{2}, {\ldots,x}_{T}$. Then $N=\sum_{t=1}^{T} x_{t}$. MUCD is the average completion days of the work units $MUCD=\frac{1}{N}\sum_{t=1}^{T} tx^{t}$. To calculate AUC, we start by calculating cumulative progress as $y_{t}=\sum_{i=1}^{t} x_{i}$. AUC is now the sum of the cumulative progress values:

$AUC=\sum_{t=1}^{T} y_{t}$, which can be evaluated as

$$AUC=\sum_{t=1}^{T} \sum_{i=1}^{t} x_{i}$$

$=\sum_{i=1}^{T} (T+1-i)x_{i}$

$=(T+1)\sum_{i=1}^{T} x_{i}-\sum_{i=1}^{T} ix_{i}$

$=(T+1)N-N\times MUCD.$

For us, *N* is fixed at 14 half-hour units, AUC is a linear transformation of MUCD, and the results in this paper involving MUCD would be identical if we used AUC instead. We chose to use MUCD due to its more intuitive psychological interpretation as the mean of the time course of work progress. Additionally, the direction of MUCD aligns with the level of procrastination, where higher values indicate more procrastination. Conversely, AUC follows the opposite direction, with higher values indicating less procrastination.

Supplementary Figure 1. (A) Histogram of hours of research participation in the last third of the semester. (B) Histogram of task starting day. (C) Histogram of day of the halfway point.

Supplementary Table 1. Pearson Correlations among metrics of procrastination

| Variables | Task completion day | Hours in the last third of the semester | Task starting day | MUCD | Day of the halfway point |
| --- | --- | --- | --- | --- | --- |
| Task completion day | 1 | 0.70 | 0.39 | 0.79 | 0.72 |
| Hours in the last third semester |  | 1 | 0.73 | 0.92 | 0.84 |
| Task starting day |  |  | 1 | 0.76 | 0.60 |
| MUCD |  |  |  | 1 | 0.93 |
| Day of the halfway point |  |  |  |  | 1 |

Note: All correlations are significant at *p* < 0.001.

**Exploratory analyses**

**Participants were aware of their own level of procrastination in research participation.** To examine whether participants were aware of their own level of procrastination in research participation, they were first asked to recall how they allocated their time throughout the semester to fulfill the research participation requirement. Then, they were asked to rate their procrastination level from not at all (1) to an extreme extent (5) to fulfill the research participation requirement. Having the recall question before the rating question is to have participants rate their procrastination level based on their recalled time course of progress in fulfilling the requirement. We found that the rating of their own procrastination level in research participation positively correlates with their behavioral level of procrastination characterized by MUCD (*r*=0.68, *p*<0.001), day of the halfway point (*r*=0.63, *p*<0.001), hours in last third semester (*r*=0.75, *p*<0.001) and task completion day (*r*=0.54, *p*<0.001). This suggests that participants were aware of their own level of procrastination in the research participation task.

**High procrastinators were less satisfied with (and regret more) the way they allocated their time over the semester to fulfill the research participation requirement.** We asked participants to rate how much they agreed with the following statement from strongly disagree (1) to strongly agree (7): I am satisfied with the way in which I allocated my time throughout the semester to fulfill the research participation requirement. We found a negative correlation between how much participants were satisfied with the way they allocated their time over the semester to fulfill the requirement and their behavioral level of procrastination quantified by MUCD (*r*=-0.57, *p*<0.001), day of the halfway point (*r*=-0.48, *p*<0.001), hours in last third semester (*r*=-0.59, *p*<0.001) and task completion day (*r*=-0.32, *p*=0.002).

We used the Regret Elements Scale[^2^](https://paperpile.com/c/AeZq7C/1Er0) to test whether high procrastinators regret more in both the cognitive domain and affective domain about how they allocate their time throughout the semester to fulfill the research participation requirement. We found a correlation between how much participants regret the way they allocated their time both in affective and cognitive domains and their behavioral level of procrastination (affective regret: procrastination quantified by MUCD (*r*=0.51, *p*<0.001), day of the halfway point (*r*=0.45, *p*<0.001), hours in last third semester (*r*=0.56, *p*<0.001) and task completion day (*r*=0.28, *p*=0.006); cognitive regret: procrastination quantified by MUCD (*r*=0.51, *p*<0.001), day of the halfway point (*r*=0.45, *p*<0.001), hours in last third semester (*r*=0.58, *p*<0.001) and task completion day (*r*=0.37, *p*<0.001).)

**Attribution of procrastination and success in fulfilling the requirement.** We used the Causal Dimension Scale[^3^](https://paperpile.com/c/AeZq7C/NpyF) to test our hypothesis that high procrastinators attribute their high procrastination to more external, temporal, and uncontrollable factors. In contrast, low procrastinators attribute their low procrastination to more internal, stable, and controllable factors. There was no evidence of a correlation between the behavioral level of procrastination and locus of causality (internal or external) (Take MUCD as an example: *r*=-0.12, *p*=0.27), stability (*r*=-0.093, *p*=0.37) and controllability (*r*=-0.17, *p*=0.11). We also used the Causal Dimension scale to test another hypothesis that high procrastinators attribute their success in fulfilling the requirement to more external, temporary, and uncontrollable factors. In contrast, low procrastinators attribute their success to more internal and stable factors. There was no evidence of a correlation between the behavioral level of procrastination and locus of causality (internal or external) (Take MUCD as an example: *r*=-0.054, *p*=0.61), stability (*r*=0.034, *p*=0.75) and controllability (*r*=-0.049, *p*=0.64).

**Top-rated procrastination reasons.** We tested self-reported reasons for procrastination in fulfilling the research participation requirement by asking the participants to rate from not at all reflects why (1) to definitely reflects why (7) on statements that consist of the four reasons: the excitement of doing things at last moment, time management, task aversiveness, and laziness. The statements are adapted from reasons for procrastination[^4^](https://paperpile.com/c/AeZq7C/H35F). The top-rated reason is time management (*M=*2.57, *SD=*1.01), followed by task aversiveness (*M*=2.19, *SD*=0.97), laziness (*M*=1.90, *SD*=0.92) and excitement of doing things at last moment (*M*=1.33, *SD*=0.64). It is important to note that ratings for all four reasons are relatively low.

**Impulsivity, self-control, and perfectionism**. Impulsivity, self-control, and perfectionism are traits associated with self-reported procrastination[^5^](https://paperpile.com/c/AeZq7C/zpXw). We examined whether these traits are correlated with behavioral procrastination in the research participation task. We found a positive correlation between impulsivity measured by the Barratt Impulsivity Scale[^6^](https://paperpile.com/c/AeZq7C/GB9p) and behavioral level of procrastination quantified by MUCD (*r*=0.26, *p*=0.013). There is no evidence of a correlation between the behavioral level of procrastination and self-control measured by the Brief Self-Control Scale[^7^](https://paperpile.com/c/AeZq7C/YNQv) (*r* =-0.14, *p*=0.18), maladaptive perfectionism (*r*=0.12, *p*=0.25), or adaptive perfectionism (*r*=-0.016, *p*=0.88). The measurement of maladaptive and adaptive perfection follows Enns et al. (2001)[^8^](https://paperpile.com/c/AeZq7C/to7Q), where maladaptive perfectionism is measured from three subscales: socially prescribed perfectionism[^9^](https://paperpile.com/c/AeZq7C/XlP5), concern over mistakes and doubts about actions[^10^](https://paperpile.com/c/AeZq7C/DWiJ), and adaptive perfectionism is measured from two subscales: self-oriented perfectionism[^9^](https://paperpile.com/c/AeZq7C/XlP5) and personal standard[^10^](https://paperpile.com/c/AeZq7C/DWiJ). Next, to test if these traits mediate the correlation between temporal discounting and procrastination, we first examined whether these traits correlate with the discount rate; showing a correlation is a necessary condition for them to be mediators. We found no evidence of a correlation between the discount rate and impulsivity (*r*=0.0022, *p*=0.98), self-control (*r*=-0.11, *p*=0.34), maladaptive perfectionism (*r*=0.13, *p*=0.24), or adaptive perfectionism (*r*=0.020, *p*=0.86).

**References**

1. [Steel, P., Svartdal, F., Thundiyil, T. & Brothen, T. Examining Procrastination Across Multiple Goal Stages: A Longitudinal Study of Temporal Motivation Theory. *Front. Psychol.* **9**, 327 (2018).](http://paperpile.com/b/AeZq7C/KA9f)

2. [Buchanan, J., Summerville, A., Lehmann, J. & Reb, J. The Regret Elements Scale: Distinguishing the affective and cognitive components of regret. *Judgm. Decis. Mak.* **11**, 275–286 (2016).](http://paperpile.com/b/AeZq7C/1Er0)

3. [Russell, D. The Causal Dimension Scale: A measure of how individuals perceive causes. *J. Pers. Soc. Psychol.* **42**, 1137–1145 (1982).](http://paperpile.com/b/AeZq7C/NpyF)

4. [Solomon, L. J. & Rothblum, E. D. Academic procrastination: Frequency and cognitive-behavioral correlates. *J. Couns. Psychol.* (1984).](http://paperpile.com/b/AeZq7C/H35F)

5. [Steel, P. The nature of procrastination: a meta-analytic and theoretical review of quintessential self-regulatory failure. *Psychol. Bull.* **133**, 65–94 (2007).](http://paperpile.com/b/AeZq7C/zpXw)

6. [Patton, J. H., Stanford, M. S. & Barratt, E. S. Factor structure of the Barratt impulsiveness scale. *J. Clin. Psychol.* **51**, 768–774 (1995).](http://paperpile.com/b/AeZq7C/GB9p)

7. [Tangney, J. P., Baumeister, R. F. & Boone, A. L. High self-control predicts good adjustment, less pathology, better grades, and interpersonal success. *J. Pers.* **72**, 271–324 (2004).](http://paperpile.com/b/AeZq7C/YNQv)

8. [Enns, M. W., Cox, B. J., Sareen, J. & Freeman, P. Adaptive and maladaptive perfectionism in medical students: a longitudinal investigation. *Med. Educ.* **35**, 1034–1042 (2001).](http://paperpile.com/b/AeZq7C/to7Q)

9. [Hewitt, P. L. & Flett, G. L. Perfectionism in the self and social contexts: conceptualization, assessment, and association with psychopathology. *J. Pers. Soc. Psychol.* **60**, 456–470 (1991).](http://paperpile.com/b/AeZq7C/XlP5)

10. [Frost, R. O., Marten, P., Lahart, C. & Rosenblate, R. The dimensions of perfectionism. *Cognit. Ther. Res.* **14**, 449–468 (1990).](http://paperpile.com/b/AeZq7C/DWiJ)
